# Single-incision laparoscopic antrectomy for type I gastric neuroendocrine tumor: a case report

**DOI:** 10.1186/s40792-021-01109-7

**Published:** 2021-01-12

**Authors:** Junya Kitadani, Toshiyasu Ojima, Keiji Hayata, Masahiro Katsuda, Shinta Tominaga, Naoki Fukuda, Hideki Motobayashi, Shotaro Nagano, Masaki Nakamura, Hiroki Yamaue

**Affiliations:** Second Department of Surgery, School of Medicine, Wakayama Medical University, 811-1 Kimiidera, Wakayama, 641-8510 Japan

**Keywords:** Gastric neuroendocrine tumor, SILS, Antrectomy

## Abstract

**Background:**

Type I gastric neuroendocrine tumors (GNETs) originate from hyperplasia of enterochromaffin­like (ECL) cells and are commonly detected in patients with chronic atrophic gastritis, including autoimmune gastritis. Typical treatment for type I GNETs comprises simple surveillance and/or endoscopic resection. For alleviation of hypergastrinemia resulting in ECL cell hypertrophy, antrectomy is a treatment option. Type I GNETs mostly have excellent prognosis, and if a surgical approach is chosen, the procedure must be minimally invasive. One such technique for multiple type I GNETs, minimally invasive single-incision laparoscopic antrectomy (SILA), is reported here for the first time.

**Case presentation:**

We performed SILA on a 46-year-old woman who developed type I GNETs caused by hypergastrinemia due to autoimmune gastritis. A Lap-Protector was inserted in a 3 cm incision at the umbilicus, and set an EZ Access equipped with two 5 mm trocars and one 12 mm trocar. Antrectomy without lymph node dissection was performed using a 5 mm forward-oblique viewing endoscope, a vessel sealing device, and linear staplers, while reconstruction was by Billroth I reconstruction. Side-to-side anastomosis was performed using a 45 mm linear stapler. The stapler entry hole was sutured intracorporeally using barbed suture material. The operation time was 140 min and blood loss was 5 ml. The patient was discharged ten days after surgery without complications. Serum gastrin level decreased to within the normal range on the day after the operation. One year after surgery, esophagogastroduodenoscopy showed pathological disappearance of all lesions of the remnant stomach.

**Conclusions:**

SILA is a minimally-invasive and tolerable technique for treatment of multiple type I GNETs. In this reported case there was good cohesiveness and effectiveness in normalizing gastrin levels and in elimination of remnant gastric lesions.

## Background

Neuroendocrine tumors (NETs) are rare neoplasms that arise from the peripheral neuroendocrine system and are dispersed in various organs [1]. Gastric NETs (GNETs) were classified into three types by Rindi et al. [2]. Type I comprise 70­−80% of all GNETs; they are composed of enterochromaffin­like (ECL) cells and are commonly detected in patients with chronic atrophic gastritis, including autoimmune gastritis and *Helicobacter pylori*-associated atrophic gastritis. Achlorhydria caused by the loss of fundic glands results in hypergastrinemia and hyperplasia of ECL cells. Most type I GNETs correspond with NET G1 in the World Health Organization (WHO) classification, with a metastasis risk of 2–5% [3]. Meanwhile, type II GNETs are associated with multiple endocrine neoplasia type 1 (MEN1) and Zollinger­Ellison syndrome and account for 5–6% of GNETs. Most type II GNETs correspond to NET G1/G2, with a metastasis risk of 10–30%. Type III GNETs comprise 10­−15% of gastric NETs; they are sporadic tumors that develop independently from gastrin secretion. They include NET G3 and neuroendocrine carcinoma (NEC), with a metastasis risk above 50%.

Regarding treatments of Type I GNETs, the National Comprehensive Cancer Network (NCCN) guidelines recommend simple surveillance or endoscopic resection for tumors < 20 mm in size and without features of invasion of muscularis propria or metastasis, regardless of the number of tumors [4]. Antrectomy is another treatment option for younger patients and cases for whom frequent follow-up endoscopy would be difficult. It alleviates G­ cell­ mediated hypergastrinemia, resulting in ECL cell hypertrophy [5]. The significance of antrectomy for Type I GNETs is controversial, the European Neuroendocrine Tumor Society (ENETS) guidelines state that its significance remains discordant [6]. We propose that laparoscopic surgery, especially single-incision laparoscopic surgery (SILS), may be suitable for the treatment of type I GNETs, which is seen predominantly in young patients. SILS offers excellent cosmetic results because scarring is concealed in the umbilicus. There are a number of risks related to surgery, however, such as anastomotic leakage, delayed gastric emptying and intraabdominal abscesses [7, 8]. There may also be short or long term decline in nutritional status. Sufficient informed consent is thus required to make a decision on performance of antrectomy.

## Case presentation

**Fig. 1:**
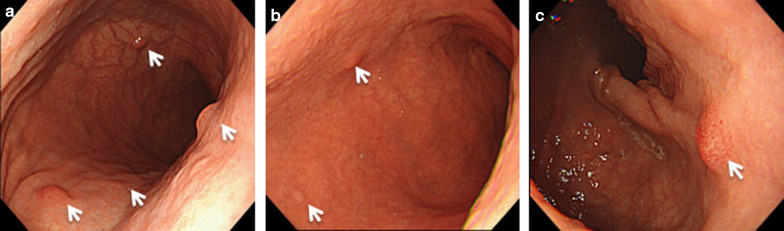
Pretreatment endoscopic findings. EGD shows an atrophic pattern and many 3–5 mm submucosal tumor-like elevated lesions around the gastric body (**a, b**) and fundus (**c**) (white arrows)

**Fig. 2:**
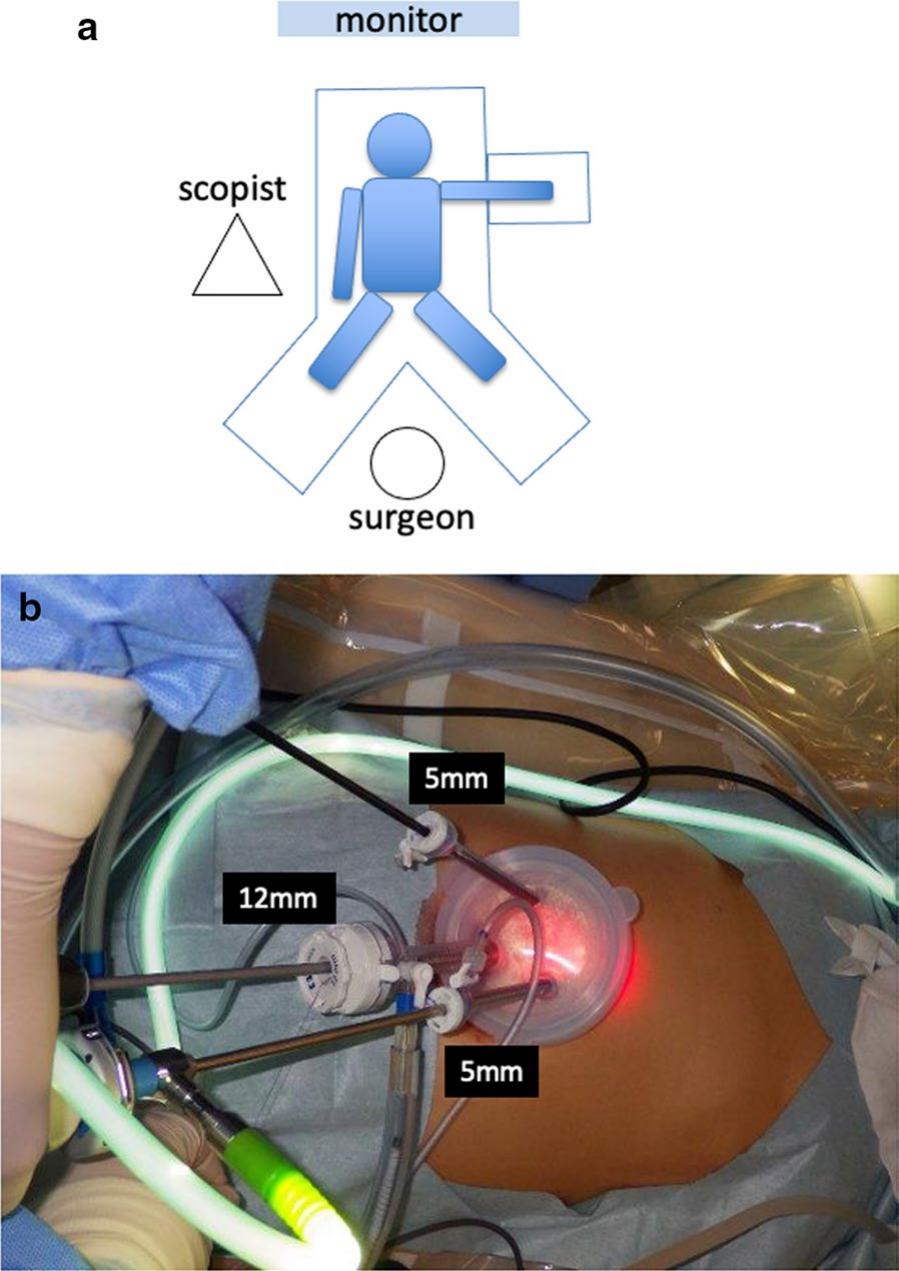
Surgical port setting. **a** The position between the monitor, the surgeon and the scopist is shown. **b** EZ Access equipped with two 5 mm trocars and one 12 mm trocar with an evacuation system for surgical smoke was set

**Fig. 3:**
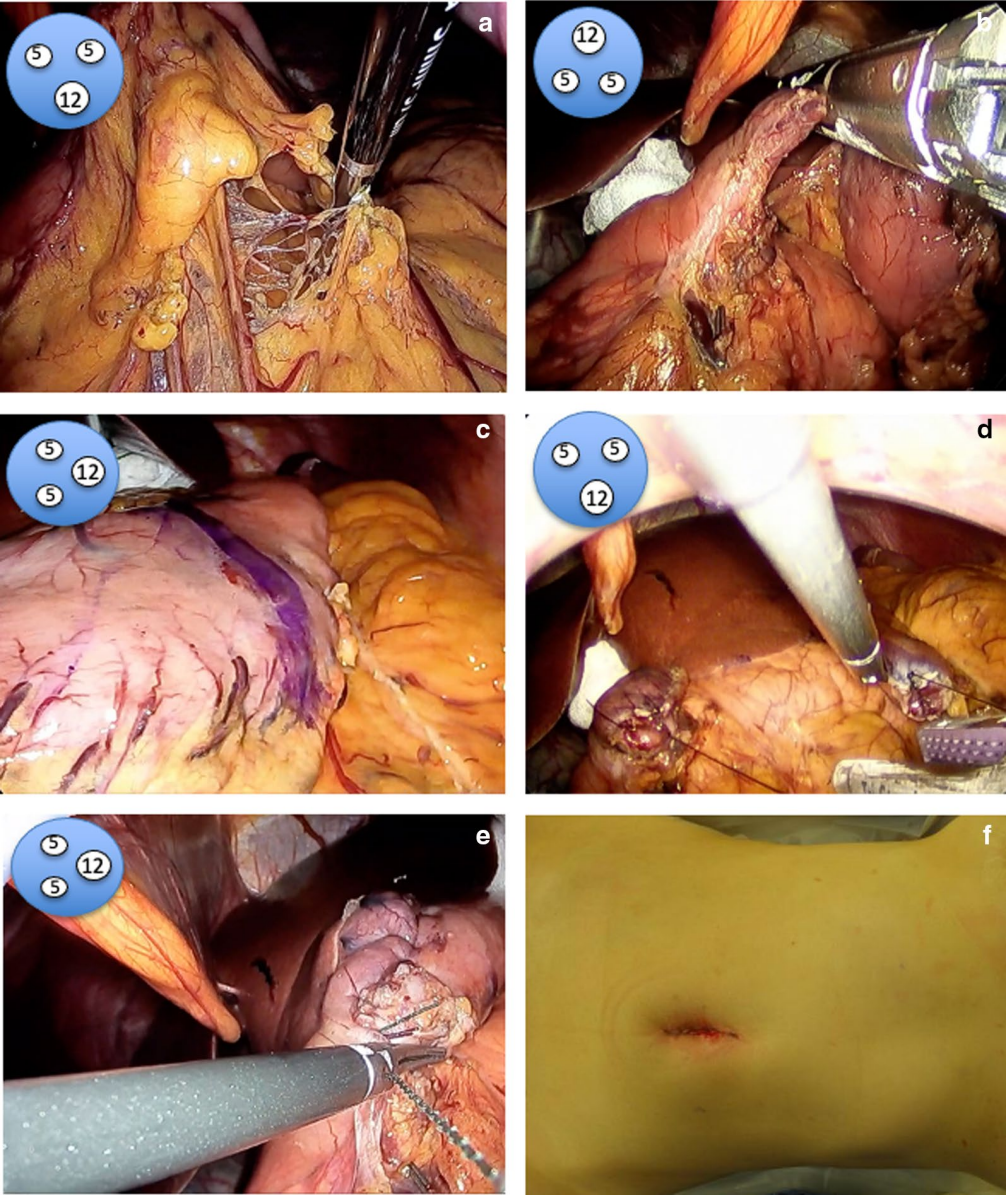
The surgical procedure of SILA. **a**–**c** Antrectomy without lymph node dissection was performed using a 5 mm forward-oblique viewing endoscope, a vessel sealing device, and linear staplers. **d** Side-to-side anastomosis was performed between posterior sides of the duodenal stump and remnant gastric stump like the delta-shaped anastomosis. **e** The entry hole was sutured intracorporeally using the barbed suture material. **f** There was only one wound, in the umbilical region. Illustrations demonstrating rotation of the EZ access port are shown in the upper left of the images

**Fig. 4:**
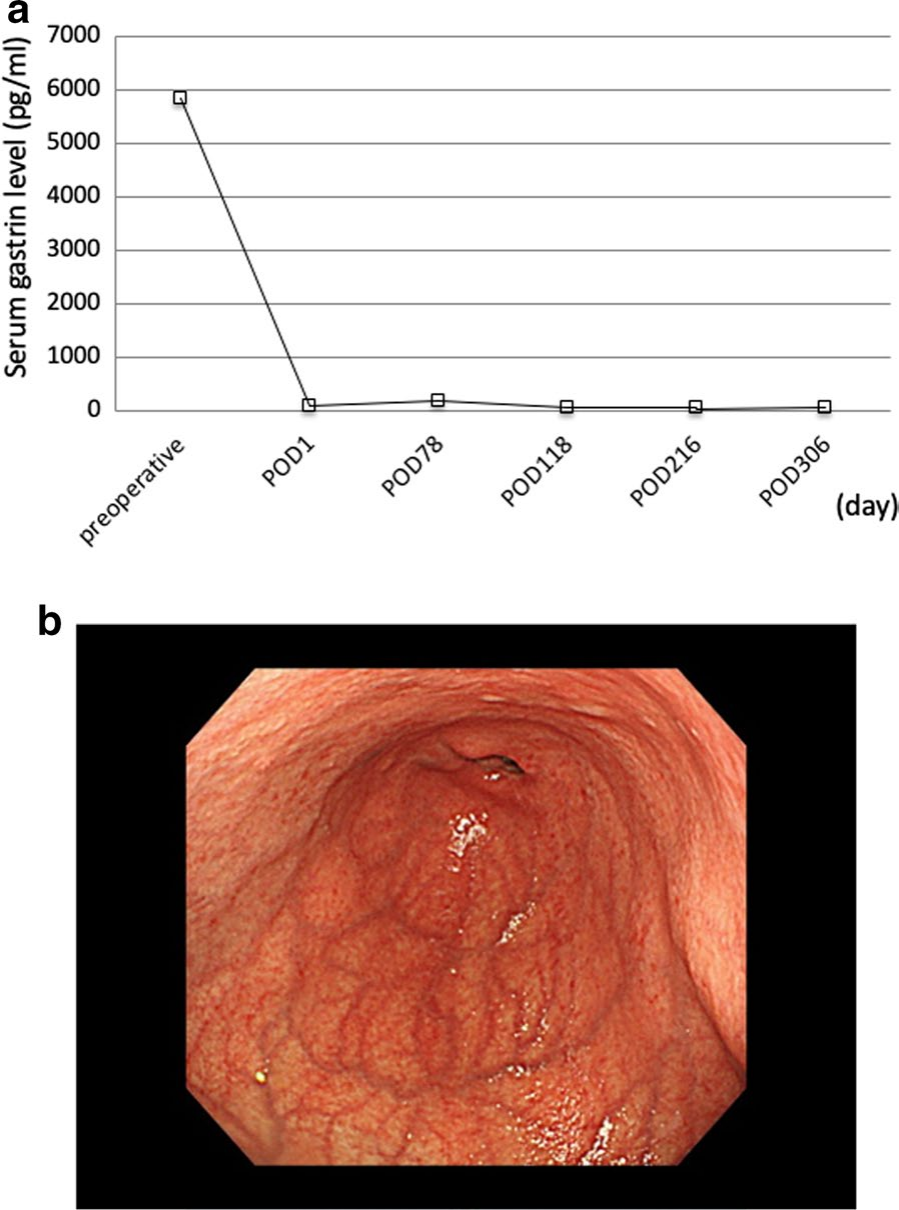
Postoperative fluctuations in gastrin levels and endoscopic findings. **a** Serum gastrin level decreased immediately within the normal range after the SILA and continued without any increase thereafter. **b** EGD performed at one year after surgery showed complete disappearance of all lesions of the remnant stomach

A 46-year-old woman had unsuccessful eradication of *Helicobacter pylori.* She had history of hypothyroidism with no particular symptoms. Esophagogastroduodenoscopy (EGD) showed chronic atrophic gastritis and numerous 3–5 mm submucosal tumor-like elevated lesions around the gastric body and fundus (Fig. 1a–c). Endoscopic ultrasonography (EUS) showed that the submucosal tumor-like lesions were commonly present in the second and third echo layers. Biopsy of those lesions showed the absence of parietal cells, but the presence of atrophic gastritis and neuroendocrine cell hyperplasia with confirmation of immunoreactivity to chromogranin A, consistent with definition of GNETs. Histologically, seven multiple tumors were found. The MIB-1 index was 1% or less, leading to diagnosis of NETs G1. Additionally, blood examinations showed serum gastrin level as high as 5850 pg/ml (standard value: 42–200 pg/ml), and anti-gastric parietal cell antibody was increased 160-fold. Abdominal contrast-enhanced computed tomography (CT) showed no lymph node metastasis or distant metastasis. We diagnosed this patient as having type I GNETs caused by hypergastrinemia due to autoimmune gastritis. Although the patient was also considered for endoscopic surveillance, we decided to perform single-incision laparoscopic antrectomy (SILA) to reduce the need for EGD follow-up. The patient was offered long-term endoscopic follow-up, and she hoped to undergo minimally invasive surgery if surgical treatment could be expected to eliminate the tumors. SILA was performed by a surgeon and a scopist in reverse Trendelenburg position. First, a 3 cm incision was made at the umbilicus under general and epidural anesthesia. A 70 × 70 mm Lap-Protector (Hakko Co., Ltd., Nagano, Japan) was then inserted, and an EZ Access (Hakko Co., Ltd., Nagano, Japan) equipped with two 5 mm trocars and one 12 mm trocar with an evacuation system for surgical smoke was set (Fig. 2a, b). Antrectomy without lymph node dissection was performed using a 5 mm 30° forward-oblique viewing endoscope, a vessel sealing device, and linear staplers (Signia Stapling System, Covidien Japan, Tokyo, Japan) (Fig. 3a–c). To adjust the angle of the device, the EZ access port equipped with three trocars was rotated as appropriate. Reconstruction was by Billroth I reconstruction. Side-to-side anastomosis was performed between posterior sides of the duodenal stump and remnant gastric stump like the delta-shaped anastomosis using 45 mm of purple cartridge Signia Stapling System (Fig. 3d). The important point is to hang two threads near the small foramen of the remnant stomach and duodenum for delta-like anastomosis and have the assistant pull them out from the 12 mm port. By this procedure, there is no tissue displacement during anastomosis. The surgeon's left forceps act to pull the duodenal stump outward. The stapler entry hole was sutured intracorporeally using the 15 cm 3–0 V-Loc 180 (Covidien, Mansfield, MA, USA), a barbed suture material (Fig. 3e). The operation time was 140 min and blood loss was 5 ml. There was only one wound in the umbilical region, so the procedure led to good cosmetic results. (Fig. 3f). The patient had a solid meal on the third day after surgery and was discharged ten days after surgery without complications. Serum gastrin level decreased to 84 pg/ml within the normal range on the day after the operation, and thereafter reverted to the normal range (Fig. 4a). Although the patient's follow-up period was still short, EGD performed at one year after surgery showed complete disappearance of all lesions of the remnant stomach, a contrast with the large number of GNETs that were scattered throughout the gastric body and fundus that were observed before the operation (Fig. 4b; Additional File 1: Video S1).

## Discussion

Type I GNETs are known to be associated with other autoimmune endocrine disorders, such as Hashimoto’s thyroiditis, premature ovarian failure, vitiligo, and diabetes mellitus type 1, as well as autoimmune gastritis [9]. This patient also had hypothyroidism. The rate of *Helicobacter pylori* infection in Type 1 GNETs is lower than the general distribution, but it is also involved in the enhancement of atrophy by autoimmune gastritis [10].

For the treatment of type I GNET, endoscopic mucosal resection (EMR) or endoscopic submucosal dissection (ESD) may be considered to be minimally invasive, as long as there are just a small number of lesions [11]. In the case of characteristic multiple lesions, however, the disorder is difficult to control by EMR or ESD. Although antrectomy has been proved to be effective in abolishing hypergastrinemia [12], subtotal gastrectomy and total gastrectomy have often been performed when there were multiple small lesions throughout the stomach [13]. The study also showed that the majority of conservatively treated patients had a higher recurrence rate according to endoscopic surveillance compared with patients who were operated upon or treated with somatostatin analogs. However, compared with endoscopic treatment, laparoscopic antrectomy can be a more invasive surgical procedure for Type I GNETs [14, 15]. Until now, however, minimally invasive SILA for multiple type I GNETs, meanwhile, has never been reported. We propose SILA as a minimally-invasive surgery with promising potential for excellent cosmesis. Some institutions have gradually begun to report their experiences of single incision distal gastrectomy (SIDG), most of which are additional SIDG, for example an assistant port is inserted, so unlike the currently described technique [16, 17]. Type I GNETs have low risk of lymph node metastasis and in this case preoperative enhanced CT showed no regional lymph node swelling, so we performed SILA without lymph node dissection. The risk of metastasis to Type I GNETs is 2–5% and there is insufficient evidence for the effectiveness of lymph node dissection. When invasion of the muscularis propria is suspected, both the ENETS and the NCCN guidelines recommend lymph node dissection. In this case, however, lymph node dissection was not performed because none of the lesions were suspected to be infiltrating the muscularis propria. The oral edge of the pyloric antrum often reaches near the cardia on the lesser curvature side, so it is necessary to resect sufficiently close to the cardia on the lesser curvature side. As previously reported, we suggest that SILA with lymph node dissection can also be safely performed similarly to conventional laparoscopic surgery [16, 17]. It is assumed, however, that an assist port would be required. Some problems, such as securing a visual field, organ exclusion, and organ pulling, can be overcome by the use of certain technical devices. It is possible to obtain a good operative field of view, for example, by rotating the EZ access and using a 5 mm forward-oblique viewing endoscope. Gauze is effective for the liver elimination. During reconstruction, pulling the thread on the remnant stomach and duodenum means side-to-side anastomosis is possible without assistance. In the currently described case, postoperative wound pain was mild and the wound was inconspicuous. Creating a tumor-free situation is reported to lead to greater level of satisfaction than having anxiety related to potential recurrence through long-term endoscopic surveillance [14].

## Conclusion

Although antrectomy may be among the most invasive methods in the treatment of type I GNETs, it may also be the most effective in terms of normalizing gastrin levels and eliminating remnant gastric lesions. We propose SILA for multiple Type I GNETs for advantages including minimal invasiveness and tolerability.

## Supplementary Information

**Additional file 1.** Supplementary Video S1: Technical video of SILA.

## Data Availability

Data sharing is not applicable to this article, as no datasets were generated or analyzed during the current study.

## Abbreviations

GNETs: Gastric neuroendocrine tumors
ECL: Enterochromaffin­like
WHO: World Health Organization
MEN1: Multiple endocrine neoplasia type 1
NEC: Neuroendocrine carcinoma
NCCN: National Comprehensive Cancer Network
ENETS: European Neuroendocrine Tumor Society
SILS: Single-incision laparoscopic surgery
EGD: Esophagogastroduodenoscopy
EUS: Endoscopic ultrasonography
CT: Contrast-enhanced computed tomography
SILA: Single-incision laparoscopic antrectomy
EMR: Endoscopic mucosal resection
ESD: Endoscopic submucosal dissection
SIDG: Single incision distal gastrectomy
